# Reciprocal Induction of MDM2 and MYCN in Neural and Neuroendocrine Cancers

**DOI:** 10.3389/fonc.2020.563156

**Published:** 2020-12-23

**Authors:** Hung N. Tran, Hardeep P. Singh, Wenxuan Guo, Linda Cambier, Luke Riggan, Gregory M. Shackleford, Matthew E. Thornton, Brendan H. Grubbs, Anat Erdreich-Epstein, Dong-Lai Qi, David Cobrinik

**Affiliations:** ^1^Division of Hematology/Oncology, Children’s Hospital Los Angeles, Los Angeles, CA, United States; ^2^The Vision Center, Department of Surgery, Children’s Hospital Los Angeles, Los Angeles, CA, United States; ^3^The Saban Research Institute, Children’s Hospital Los Angeles, Los Angeles, CA, United States; ^4^Department of Ophthalmology and Roski Eye Institute, Keck School of Medicine, University of Southern California, Los Angeles, CA, United States; ^5^Program in Biomedical and Biological Sciences, Keck School of Medicine, University of Southern California, Los Angeles, CA, United States; ^6^Department of Radiology, Children’s Hospital Los Angeles, Los Angeles, CA, United States; ^7^Department of Obstetrics and Gynecology, Keck School of Medicine, University of Southern California, Los Angeles, CA, United States; ^8^Departments of Pediatrics and Pathology, Children’s Hospital Los Angeles and Keck School of Medicine, and Norris Comprehensive Cancer Center, Keck School of Medicine, University of Southern California, Los Angeles, CA, United States; ^9^Medicine and Pharmacy Research Center, Binzhou Medical University, Yantai, China; ^10^Department of Biochemistry and Molecular Medicine and Norris Comprehensive Cancer Center, Keck School of Medicine, University of Southern California, Los Angeles, CA, United States

**Keywords:** MDM2, MYCN, MYC, neuroblastoma, retinoblastoma, small cell lung cancer, medulloblastoma

## Abstract

MYC family oncoproteins MYC, MYCN, and MYCL are deregulated in diverse cancers and via diverse mechanisms. Recent studies established a novel form of MYCN regulation in MYCN-overexpressing retinoblastoma and neuroblastoma cells in which the MDM2 oncoprotein promotes MYCN translation and MYCN-dependent proliferation via a p53-independent mechanism. However, it is unclear if MDM2 also promotes expression of other MYC family members and has similar effects in other cancers. Conversely, MYCN has been shown to induce MDM2 expression in neuroblastoma cells, yet it is unclear if MYC shares this ability, if MYC family proteins upregulate MDM2 in other malignancies, and if this regulation occurs during tumorigenesis as well as in cancer cell lines. Here, we report that intrinsically high MDM2 expression is required for high-level expression of MYCN, but not for expression of MYC, in retinoblastoma, neuroblastoma, small cell lung cancer, and medulloblastoma cells. Conversely, ectopic overexpression of MYC as well as MYCN induced high-level MDM2 expression and gave rise to rapidly proliferating and MDM2-dependent cone-precursor-derived masses in a cultured retinoblastoma genesis model. These findings reveal a highly specific collaboration between the MDM2 and MYCN oncoproteins and demonstrate the origin of their oncogenic positive feedback circuit within a normal neuronal tissue.

## Introduction

MYC family proteins MYC, MYCN, and MYCL have important roles in development, homeostasis, and oncogenesis (1, 2). During development, the different MYC family members have distinct expression patterns (2, 3) yet share the ability to promote growth of the tissues in which they are expressed (4). The contributions of particular MYC family proteins in different cancers parallel their developmental expression, with deregulation of MYC in diverse hematologic and solid tumors, deregulation of MYCN mainly in neuroendocrine and neuronal tumors, and deregulation of MYCL in small cell lung carcinoma (SCLC) (5).

Whereas individual cancers typically have high-level expression of only one MYC family member, some cancers are driven by different overexpressed MYC family proteins in different patients. For example, ~19% of high-risk neuroblastomas have *MYCN* amplification and high MYCN expression, whereas ~11% overexpress MYC and less than 0.5% highly express both (6). Likewise, different SCLCs have amplified *MYC*, *MYCN*, or *MYCL* (7), and different medulloblastoma subgroups overexpress either *MYC* or *MYCN* but not both (8, 9). The largely mutually exclusive overexpression of MYC family members is consistent with their having similar oncogenic effects.

While cancers that are driven by deregulated MYC family proteins depend on such proteins for cell proliferation and survival (10, 11), it has not been possible to directly inhibit MYC family functions via small molecule therapeutic agents (12). As such, understanding and targeting the mechanisms that drive MYC family overexpression is an attractive alternative approach (5, 13, 14). We and others have reported that MDM2 drives MYCN overexpression and MYCN-dependent proliferation in neuroblastoma and retinoblastoma cells by enhancing its translation in a p53-independent manner (15–17). Specifically, MDM2 was found to upregulate *MYCN* RNA levels and *MYCN* translation in NB1691 and SK-N-SH neuroblastoma cells and in RB176 and Y79 retinoblastoma cells, effects attributed to *MYCN* RNA stabilization and ribosomal loading in the neuroblastoma setting (15, 17). The high MDM2 levels needed to drive MYCN expression exceed those needed to suppress p53-mediated apoptosis and thus abrogate the need for genomic inactivation of p53-mediated tumor surveillance signaling in retinoblastoma and neuroblastoma tumors (16, 17).

Despite the importance of the MDM2-MYCN axis in neuroblastoma and retinoblastoma, it has been unclear if MDM2 promotes MYCN expression in additional cancers and if MDM2 also regulates MYC. Furthermore, factors that drive high MDM2 expression preceding its induction of MYCN are not well understood. In retinoblastoma cells, *MDM2* transcription is in part driven by RXRγ, a cell lineage transcription factor in the cone precursor cell of origin (18, 19), yet the basis for further MDM2 upregulation in retinoblastoma tumors has not been established. In *MYCN*-amplified neuroblastoma cell lines, MYCN itself transcriptionally activates *MDM2* (20), suggestive of a MYCN-MDM2 positive feedback circuit, yet it is unclear if MYCN or MYC upregulates MDM2 in autochthonous tumor initiation.

Here, we investigated whether MDM2 upregulates MYC as well as MYCN in selected neural and neuroendocrine cancers. We also assessed whether ectopic MYC and MYCN induce MDM2 in a manner that is critical to the onset of tumorigenesis in an intact retina model of retinoblastoma initiation (21). We report that MYC and MYCN show consistently different dependence on MDM2 in cancer cell lines, yet similar abilities to induce MDM2 expression and MDM2-dependent proliferation in the retinoblastoma cell of origin. These findings provide insights into the origin and specificity of the MYCN and MDM2 interplay at the onset of a MYCN-dependent tumor.

## Materials and Methods

### Cell Lines and Cell Culture

RB176 and MYCN- or MYC-transformed cone precursors were cultured in RB medium (18). SK-N-BE(1) (22), CHLA255 (23), and SH-SY5Y (24) were provided by R. Seeger (University of Southern California), grown in RPMI with 10% fetal bovine serum (FBS) and 1% penicillin-streptomycin (PS). H69 and H526 cells were provided by I. Offringa (University of Southern California), and H211 and H82 were purchased from American Type Culture Collection. SCLCs were grown in RPMI with 10% FBS and 1% PS. DAOY, UW-228-2 (provided by J. Silber, University of Washington), D283Med, D341Med, and D425Med (provided by D. Bigner, Duke University) (25) were grown in IMDM with 20% FBS and 1% PS. Cell lines were typed for short tandem repeats and mycoplasma tested before use. Following informed consent, fetal eyes were obtained from authorized sources with approval by the University of Southern California and Children’s Hospital Los Angeles Institutional Review Boards. Fetal age was determined and retinal tissue obtained and cultured as described (21).

### DNA Constructs

Lentiviral constructs pLKO.1-sh*SCR*, -sh*MDM2-1*, -sh*MDM2-2*, and -sh*TP53*, as well as *BE-Neo* (*BN*) and *BN-MYCN* were as described (17). *BE-GFP-MYCN* was produced by inserting MYCN sequences 734–2139 (NM_001293228.1, isoform 1) into the SalI and XbaI site of the *Bidirectional EF1α-Green Fluorescent Protein* (*BE-GFP*) lentiviral vector (26) using In-Fusion (Clontech). *BE-GFP-MYCC* was produced by inserting *MYC* sequences 409–1728 (NM_002467.6, isoform 1) between *BE-GFP* SalI and XbaI sites using In-Fusion.

### Lentivirus Production and Infection

pLKO.1-sh*SCR*, -sh*MDM2-1*, -sh*MDM2-2*, and -sh*TP53* lentiviruses were produced by reverse transfection of Lenti-X cells (Clontech) as described (17) except using 17.5 μg pLKO.1 vector, 4.38 μg pVSV-G, 8.75 μg pMDL, 4.38 μg pREV, and 105 µl 0.6 μg/μl polyethylenimine per 15 cm dish for cancer cell lines and 5 μg pVSV-G, 10 μg pMDL, 5 μg pREV, and 160 µl 0.6 μg/μl polyethylenimine per dish for cone precursors. Supernatants were collected at 48 h post-transfection and filtered through 0.45 μm filter and used directly or concentrated by centrifugation (17). sh*MDM2-1*, sh*MDM2-2*, and sh*SCR* infections used 5 ml unconcentrated virus for 5 × 10^5^ cells. For co-knockdown, 100 μl concentrated sh*TP53* supernatant was combined with 5 ml unconcentrated supernatant before infections, with 4 μg/ml polybrene (Sigma-Aldrich) and pipetted 20 times. After 4–6 h, virus-infected cells were diluted with 8 ml medium. Infected cells were selected starting 48–50 h post-infection with 1–2 μg/ml puromycin for up to 48 h for cancer cell lines or 2.5 μg/ml puromycin for 28 h for cone precursors. *BN*- and *BN-MYCN* transduction and selection of SH-SY5Y cells was as described (17). *BE-GFP-MYCN* and *BE-GFP-MYCC* lentivirus were produced, concentrated, and used to transduce developing retina as described (21).

### *MYCN*- and *MYC*-Transformed Cell Derivation and Growth

Cells were derived from *MYCN*- or *MYC*-transduced retinae by gentle agitation of masses on retina surfaces and removal to RB medium (21). 70% of media in each well was refreshed when it became orange, and cells were split when media turned orange within 24 h. For growth assays, 1×10^4^ cells were seeded in 250 μl in 96-well plates separately and in triplicate (technical replicates) for each time point. Half of media in each well was refreshed daily and live cells or live/dead cell ratios counted by trypan blue staining.

### Immunoblotting

Cell lysates were prepared as described (17). Primary antibodies were MDM2: 4H26L4 (Invitrogen, Carlsbad, CA; 700555) 1:300, MYCN: NCMII100 (Santa Cruz) 1:200, p53: FL393 (Santa Cruz) 1:300, p21: C-19 (Santa Cruz) 1:100; α-tubulin: B 5-1-2 (Sigma) 1:5000; and HRP-conjugated secondary antibodies (Santa Cruz). Densitometry was performed with ImageJ.

### Quantitative Immunostaining

Retinal culture, transduction, and immunostaining were as described (21). Immunostaining antibodies were: Ki67: 550609 (BD), 1:200; MDM2: SC-965 (Santa Cruz), 1:150; MYCN: SC56729 (Santa Cruz) 1:100; MYC: D84C12 (Cell Signaling Technology) 1:800; RXRG: SC-555 (Santa Cruz) 1:1000, SC-514134 (Santa Cruz) 1:200. MDM2 expression was quantitated using Trainable Weka Segmentation plugin available in Fiji (27) (https://imagej.net/Trainable_Weka_Segmentation) trained to recognize and segment nuclei stained with 4′,6-diamidino-2-phenylindole (DAPI). Trained data was applied to confocal images of fluorescent antibody stained sections to obtain probability maps followed by watershed and segmentation of DAPI+ nuclei. Mean gray values of fluorochromes were measured from single cells using Fiji. Sample sizes were chosen based on availability. All segmented nuclei were included. Cell culture groups were compared using two‐tailed, unpaired, non-parametric Mann-Whitney U test. Transduced retinae groups were compared using one‐way analysis of variance (ANOVA) Kruskal-Wallis test followed by Dunn’s multiple comparisons test. GFP^Lo^ and GFP^Hi^ cells were distinguished based on mean GFP pixel density relative to 7% of the maximum in *MYCN-* and *MYCC*-transduced tissues and cell lines.

### Combined Hybridization Chain Reaction (HCR) *In Situ* Hybridization + Immunostaining

DNA probes targeting human *MYCN* coding sequence or the BE-GFP 3’ untranslated region (UTR) *WPRE* sequences were designed using Stellaris Probe Designer RNA FISH (LGC Biosearch Technologies) according to: 20 nucleotide (nt) length, spacing between each probe: 4/5 nt, masking level: 5; and purchased from Integrated DNA Technologies. Probes were 60 nt length including a 20 nt RNA-targeting sequence at 5’ end, a 4 nt linker, and a 36 nt initiator at 3’end according to the HCR amplifier system (28) (*MYCN*: B2 system-Alexa-594, *WPRE*: B1 system-Alexa-647) (**Table S1**). All solutions for HCR were prepared with diethyl pyrocarbonate (Sigma D5758)-treated and autoclaved water. Probing was performed as described (28) with minor modifications. In brief, fetal retina frozen section slides were warmed and dried at room temperature (RT) for 15 min. Sections were post-fixed with 4% paraformaldehyde (Sigma-Aldrich, #47608) in 1× phosphate buffered saline (PBS) for 10 min, washed three times with 1× PBS, and permeabilized with 0.5% sodium dodecyl sulfate (Ambion, #AM9822) in 1× PBS for 10 min, followed by three washes in 1× PBS. Slides were treated with 0.5-1 ml of 1% NaBH_4_ (Sigma-Aldrich, #452882) in 1× PBS for 5 min to reduce autofluorescence, followed by 3 × 10 min washes with 5× SSCT (sodium chloride sodium citrate (Invitrogen, #15557044), 0.1% Tween-20 (Sigma-Aldrich, #T1379)). Sections were then blocked with 1 μM random 60 nt DNA oligomers in Probe Hybridization buffer (10% Dextran Sulphate (Sigma-Aldrich, #67578), 30 % Formamide (Calbiochem, #344206), 2× SSCT) for 1 h at 40°C, and then hybridized with 1 nM pooled probe solution in probe hybridization buffer overnight at 40°C (0.1 μM probe solution in 2× SSCT was heated at 95°C for 90 s and cooled for 30 min at RT before hybridization). On the next day, slides were washed three times at RT in wash buffer (30% formamide, 2× SSCT) for 10 min each, followed by two washes in 5× SSCT for 10 min. For each probed mRNA, 6 μl of the appropriate 3 μM fluorescently labeled hairpins (Molecular Instruments, Inc.) were mixed with 10 μl of 2× sodium chloride sodium citrate, heated at 95°C for 90 s, cooled to RT for 30 min in the dark, and then mixed with 500 μl of amplification buffer (10% dextran sulfate, 2× SSCT). Slides were incubated with hairpin amplifiers in amplification buffer at RT for 1.5–2 h in the dark. After three washes in wash buffer for 10 min and 2 washes in 5× SSCT for 10 min, DNA was stained with 0.5 μg/ml DAPI in 5× SSCT for 10 min. Slides were mounted with Mowiol (Calbiochem, 475904) and imaged on a Zeiss LSM 710 confocal microscope. For immunofluorescence, coverslips were removed in PBS and sections immunostained as above. *In situ* hybridization and immunofluorescence images were merged in ImageJ (29).

## Results

### MDM2-Dependent Expression of MYCN but Not MYC in Neuroendocrine Cancers

To assess whether MDM2 has a general role in regulation of MYC family proteins, we queried the effects of MDM2 knockdown in a series of neuroendocrine cancer cell lines. We focused on cancers that have high-level expression of MYCN and/or MYC in different instances, including retinoblastoma cells that co-express MYCN and MYC (RB176), neuroblastoma cells that highly express either MYCN (SK-N-BE(1)) or MYC (CHLA255, SH-SY5Y), SCLC cells that highly express either MYCN (H69, H526) or MYC (H211, H82), and SHH-like (DAOY, UW228-2) and Group 3 (D283, D341, D425) medulloblastoma lines for which only MYC-expressing lines are known (**Table 1**).

**Table 1:** Cell lines used in this study, with each line’s expression of MYCN or MYC, *TP53* status, and MYCN or MYC dependence on MDM2 expression.

	Cell line	MYCN	MYC	*TP53*	MDM2-dependence
**Retinoblastoma**	RB176	✓	✓	wt	✓ (MYCN only)
	SK-N-BE(1)	✓		wt	✓
**Neuroblastoma**	CHLA255		✓	wt	x
** **	SH-SY5Y		✓	wt	x
	H69	✓		Mut	✓
**Small cell lung cancer**	H526	✓		Mut	✓
** **	H211		✓	Mut	x
** **	H82		✓	Mut	x
	DAOY		✓	Mut	x
** **	UW228-2		✓	Mut	x
**Medulloblastoma**	D283		✓	wt	x
** **	D341		✓	wt	x
** **	D425		✓	wt	x

**Figure 1:**
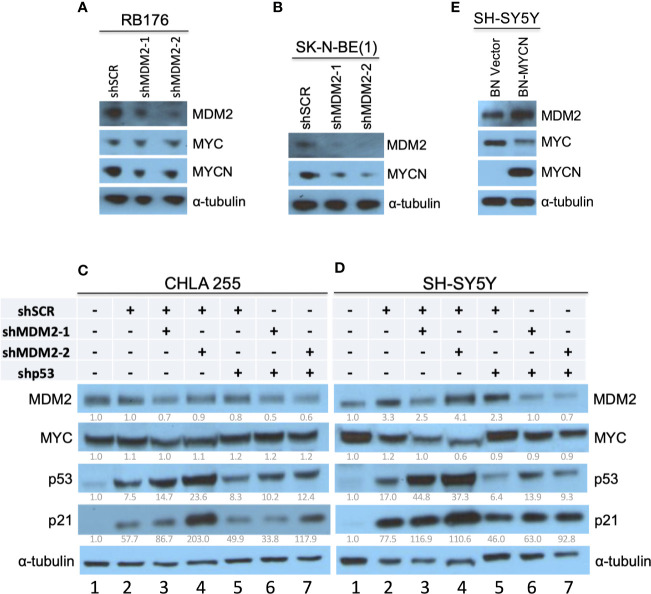
Downregulation of MYCN but not MYC in MDM2-depleted retinoblastoma and neuroblastoma cells. **(A, B)** Western analysis of MDM2, MYCN, and/or MYC in MYC and MYCN-expressing and p53 WT RB176 retinoblastoma cells **(A)** and SK-N-BE(1) neuroblastoma cells **(B)** after lentiviral transduction of sh*MDM2-1*, sh*MDM2*-2, or the sh*SCR* control. **(C, D)** Western analysis of MDM2, MYC, p53, and p21 in MYC overexpressing, *TP53* wild type CHLA 255 and SH-SY5Y neuroblastoma cells transduced as in **(A)** with additional transduction of sh*SCR* or sh*TP53* and analyzed 40 h post transfection. Relative expression normalized to α-tubulin is shown below each blot. **(E)** Western analysis of MDM2, MYC, and MYCN in SH-SY5Y cells stably transduced with *BN-MCYN* or the empty *BN* lentiviral expression vector. Immunoblots were probed for α-tubulin as loading control.

In studies establishing that MDM2 regulates MYCN in retinoblastoma (17), we noticed that retinoblastoma cell line RB176 expressed MYC in addition to MYCN. Thus, we assessed whether MDM2 regulates MYC as well as MYCN in this line by lentivirus-mediated transduction of *MDM2*-directed shRNAs (sh*MDM2-1* and *shMDM2-2*) or a scrambled control (sh*SCR*), followed by western blotting of MDM2, MYCN, and MYC. At four days after transduction, sh*MDM2*-transduced cells had lower but still detectable MDM2 and MYCN but no change in MYC (**Figure 1A**), implying that high MDM2 was needed to sustain high-level MYCN but not MYC expression.

MDM2 was also reported to be necessary for MYCN expression in *MYCN*-amplified neuroblastoma cell lines NB-1691 and LA1-55N, which are *TP53-*wild type and *TP53-*null, respectively (15). Extending this finding, we found that MDM2 knockdown downregulated MYCN in the *MYCN-*amplified and *TP53* wild type (30) cell line SK-N-BE(1) (**Figure 1B**).

Whereas *MYCN* is amplified and highly expressed in about one-third of high-risk neuroblastomas, MYC is highly expressed in a distinct subset of Stage 4 neuroblastomas that have similarly aggressive clinical behavior (6). The CHLA 255 and SH-SY5Y cell lines are representative of MYC-overexpressing and *TP53* wild type neuroblastoma cells (23, 24), and at least SH-SY5Y is MYC-dependent (31). To determine if MDM2 is needed for high MYC expression, we subjected CHLA 255 and SH-SY5Y cells to shRNA-mediated MDM2 depletion. In contrast to MYCN-overexpressing cell lines, CHLA 255 and SH-SY5Y were unable to tolerate MDM2 loss for the time needed to evaluate the MDM2 knockdown effects. At 40 h after infection, sh*MDM2*-transduced CHLA 255 and SH-SY5Y cell numbers dramatically decreased, associated with increased p53 and p21^CDKN1A^ (**Figures 1C, D**, lanes 2–4), suggestive of p53-mediated cell death. To mitigate such effects, we examined MDM2 knockdown in conjunction with p53 co-knockdown. At 40 h after transduction, this enabled downregulation of MDM2 with little induction of p53, p21, or cell death, and revealed no effect on MYC levels (**Figures 1C, D**, lanes 5–7). Thus, in contrast to MDM2’s contribution to MYCN expression, MDM2 did not contribute to MYC expression in MYC-dependent SH-SY5Y and CHLA 255 cells.

**Figure 2:**
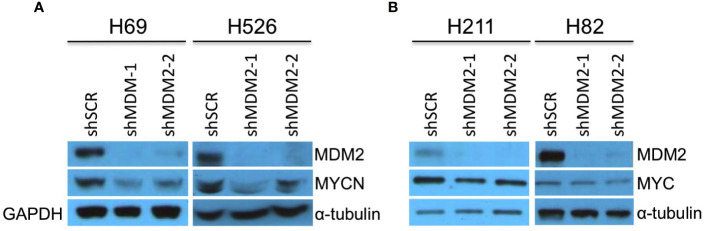
Downregulation of MYCN but not MYC in MDM2-depleted SCLC cells. Western analysis of MDM2, MYCN, and MYC expression in MYCN overexpressed, p53 mutant, SCLC lines H69 and H526 at 63 h **(A)** and in MYC overexpressed, p53 mutant SCLC lines H211 and H82 SCLC at 92 and 50 h, respectively **(B)** after lentiviral transduction of sh*MDM2-1* or sh*MDM2*-2 or sh*SCR* control. Immunoblots were re-probed for GAPDH (H69) or for α-tubulin (H526, H211, and H82) as loading controls.

SCLCs can have amplification and high-level expression of MYC, MYCN, or MYCL (7). To assess if MDM2 regulates MYC proteins in such cancers, we examined effects of MDM2 knockdown in the MYCN-overexpressing SCLC cell lines H69 and H526 and the MYC-overexpressing H211 and H82, which are all *TP53* mutant (32–35). In H69 and H526 cells, we observed that transduction of sh*MDM2* decreased MDM2 as well as MYCN expression (**Figure 2A**). In contrast, MDM2 knockdown did not substantially affect MYC in H211 or H82 cells (**Figure 2B**). Thus, as in retinoblastoma and neuroblastoma, high MDM2 was needed to maintain high-level MYCN but not MYC expression in SCLC cell lines.

**Figure 3:**
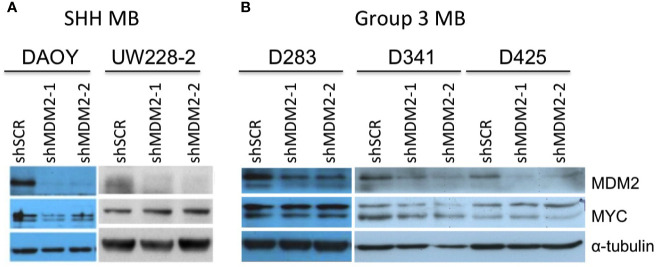
MDM2-independent expression of MYC in SHH and Group 3 medulloblastomas. Western analysis of MDM2, MYC, and α-tubulin expression in MYC overexpressed, p53 WT, SHH medulloblastoma cell lines DAOY and UW-228-2 **(A)**, and Group 3 medulloblastoma cell lines D283Med, D341Med, and D425Med cell lines **(B)** at 4 days post-transduction. Immunoblots were probed for α-tubulin as loading control.

Medulloblastomas can be divided into WNT, sonic hedgehog (SHH), Group 3, and Group 4 molecular subgroups (8). Although most SHH medulloblastomas highly express *MYCN*, the few SHH (or SHH-like) medulloblastoma cell lines (UW-228-2 and DAOY) prominently express only MYC (36, 37), similar to most Group 3 medulloblastomas (8, 38, 39). Thus, we examined effects of MDM2 knockdown on MYC expression in SHH-like DAOY and UW-228-2 as well as in *TP53* wild type Group 3 medulloblastoma cell lines D283Med, D341Med, and D425Med (25, 40, 41). At 4 days after transduction, MDM2 knockdown did not affect MYC expression in these cell lines (**Figure 3**).

### MYCN- and MYC-Dependent Upregulation of MDM2 in the Retinoblastoma Cell of Origin

The above studies revealed that MDM2 upregulates MYCN but not MYC in several neural and neuroendocrine cancers. However, the basis for the high MDM2 that drives MYCN expression in such cancers has been unclear. Prior studies showed that MYCN can drive MDM2 expression in neuroblastoma cells (20), which we corroborated in SH-SY5Y neuroblastoma cells with ectopic MYCN overexpression (**Figure 1E**). However, MYCN knockdown did not downregulate MDM2 in two of three *MYCN* amplified neuroblastoma cell lines (16, 20) or in RB176 (17), a retinoblastoma line in which MDM2 promotes MYCN expression (**Figure 1A**). Thus, we hypothesized that MYCN overexpression might initiate high-level MDM2 expression in early stages of MYCN-induced tumorigenesis despite not being required to sustain high MDM2 in MYCN-overexpressing cell lines.

**Figure 4:**
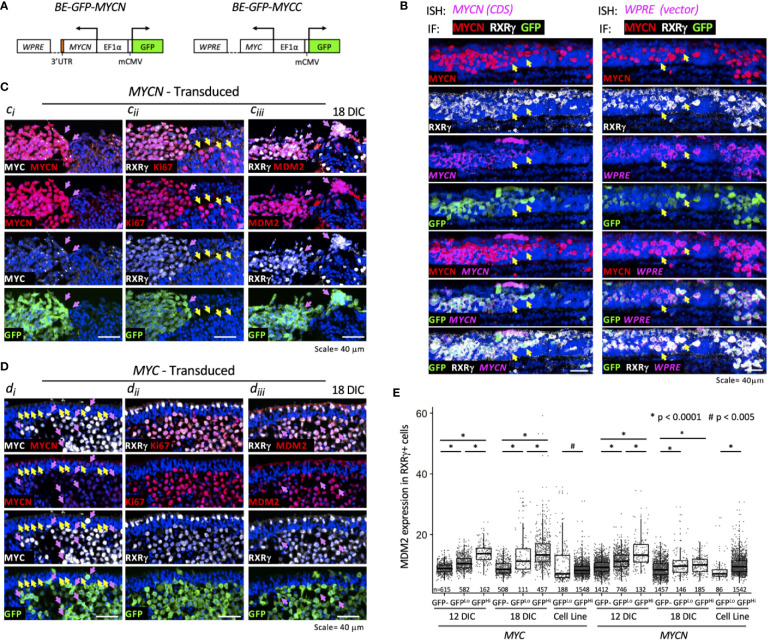
MYCN and MYC upregulate sustained MDM2 expression and proliferation in the retinoblastoma cell of origin. Cultured fetal retinae (gestational week 17.6) were transduced with *BE-GFP-MYCN* or *BE-GFP-MYCC* **(A)** and RXRγ^+^ cone precursors examined at the indicated days in culture (DIC) for expression of MYCN, MYC, MDM2, and Ki67. **(B)** Combined *in situ* hybridization (ISH) probing for *MYCN* coding sequences (CDS, left) or lentiviral vector *WPRE* sequences (right) and immunofluorescence staining (IF) for MYCN, RXRγ, and GFP. Two GFP+ cells are indicated in each panel (arrows). *MYCN* RNA, *WPRE* RNA, and MYCN protein were detected mainly in transduced GFP+,RXRγ+ cells. **(C)** Serial sections of peripheral region of an explanted retina at 18 days post-*MYCN* transduction show a mass of GFP+ cells with high MYCN **(Ci)**, RXRγ and Ki67 **(Cii)**, and MDM2 **(Ciii)** (pink arrows). Additional Ki67+ cells were GFP(-) and deemed to be retinal progenitor cells (**Cii**, yellow arrows). **(D)** Maturing central region of retinal explant at 18 days post-*MYC* transduction shows a mass of GFP+ cells with high MYC (**Di**, pink arrows), RXRγ and Ki67 (**(Dii)**, and MDM2 **(Diii)** (pink arrows). Endogenous MYCN is detected at lower levels in untransduced GFP(-) cone precursor cells (yellow arrows) and in the GFP+ mass (pink arrows). **(E)** Quantitation of MDM2 in RXRγ+ cells in 12 and 18 DIC *MYC*- and *MYCN*-transduced retinae and in derived cells cultured for an additional 30 days. Dots indicate MDM2 expression of RXRγ+ cells in three to four sections analyzed with the same staining, imaging, and quantitation parameters at each time point. Box and whisker plots show median, upper and lower quartiles (box borders), and 1.5× the interquartile range (whiskers) for GFP(-), GFP^Lo^, and GFP^Hi^ cells. n, number of cells analyzed. *P < 0.0001; ^#^P < 0.005. Data is representative of at least two separately transduced retinae.

To determine if MYCN overexpression upregulates MDM2 in early tumorigenesis, we used an intact cultured retina model that resembles *MYCN*-amplified retinoblastoma (Singh et al., in preparation). In this model, a bidirectional lentiviral vector *BE-GFP-MYCN* (**Figure 4A**) is used to coordinately express GFP and MYCN in cultured fetal retina. *BE-GFP-MYCN* transduction induces expression of numerous foci consisting of GFP+,RXRγ+ cone precursors with high-level MYCN and Ki67 expression, indicative of incipient MYCN-induced pre-retinoblastoma masses (Singh et al., in preparation). Although endogenous MYCN is far more highly expressed in human maturing cone precursors than other retinal cell types (18), it is dramatically higher and similar to that of *MYCN*-amplified retinoblastoma tumors, in *BE-GFP-MYCN*-induced foci (Singh et al., in preparation). The focal high-level MYCN in this setting reflects expression of the *BE-GFP-MYCN* vector, as combined fluorescence *in situ* hybridization plus immunostaining revealed similar high expression of *MYCN* coding sequences and vector-specific *WPRE* sequences in the transduced MYCN^Hi^,GFP+,RXRγ+ cone precursors (**Figure 4B**).

To assess the effects of ectopic *MYCN* on the endogenous MDM2 expression, an intact gestational week 17.6 retina was transduced with *BE-GFP-MYCN*, and one-half of the retina was removed and examined at 12 days in culture (DIC) and the other half examined at 18 DIC (**Figure 4C**). In parallel, the fellow retina was examined for effects of ectopic *MYC* (**Figure 4D**). *MYCN* induced numerous RXRγ+ cone precursor foci with high-level MYCN and Ki67 expression at 12 DIC, and enlarged foci by 18 DIC (**Figures 4Ci, Cii**, pink arrows). Ki67 was also detected in GFP(-) non-transduced cells (**Figure 4Cii**, yellow arrows) indicative of retinal progenitor cell proliferation (21). Notably, the MYCN^Hi^ cell population identified by immunostaining strongly induced MDM2 expression in the same retinal regions (**Figure 4Ciii**, pink arrows). Automated image analyses of GFP, RXRγ, and MDM2 co-staining across entire 12 DIC and 18 DIC retinal sections revealed that GFP+,RXRγ+ transduced cone precursors had significantly higher MDM2 than neighboring untransduced GFP(-) cone precursors (**Figure 4E**). In contrast, we detected no MDM2 upregulation in GFP+,RXRγ+ cone precursors of a retina that was transduced with the *BE-GFP* vector control, indicating that MDM2 was induced by MYCN and not by lentiviral transduction *per se*. Quantitative immunostaining revealed that MYCN upregulated MDM2 at 12 and 18 DIC (**Figure 4E**) and in another retina examined at 14 DIC. At 12 DIC, MDM2 levels were higher in GFP^Hi^ cells than GFP^Lo^ cells (**Figure 4E**), likely reflecting coordinated lentiviral expression of GFP and MYCN and MYCN-induced upregulation of MDM2. However, this trend was not evident at 18 DIC, possibly reflecting selection against the highest MYCN- and MDM2-expressing cells.

We next examined whether ectopically expressed MYC can induce cone precursor proliferation and MDM2 expression. Transduction of human *MYC* cDNA using *BE-GFP-MYCC* (**Figure 4A**), in parallel with the *MYCN* transduction in the contralateral retina, induced MYC and Ki67 expression in GFP+,RXRγ+ cone precursors at 12 and 18 DIC (**Figures 4Di, Dii**). Notably, the GFP+,RXRγ+,MYC+ cells retained intrinsic lower-level MYCN expression (**Figure 4Di**, pink arrows) similar to that of untransduced cone precursors in the outer nuclear layer (**Figure 4Di**, yellow arrows), consistent with the established MYCN expression during cone precursor maturation (18). Similar to *MYCN*-transduced cells, *MYC*-transduced cells had increased MDM2 expression (**Figure 4Diii**), with higher levels in GFP^Hi^ than GFP^Lo^ cells at both 12 and 18 DIC (**Figure 4E**). Quantitative imaging of *MYC*- and *MYCN*-transduced retina that were cultured and analyzed in parallel revealed that MYC and MYCN oncoproteins induced similar MDM2 expression (**Figure 4E**).

**Figure 5:**
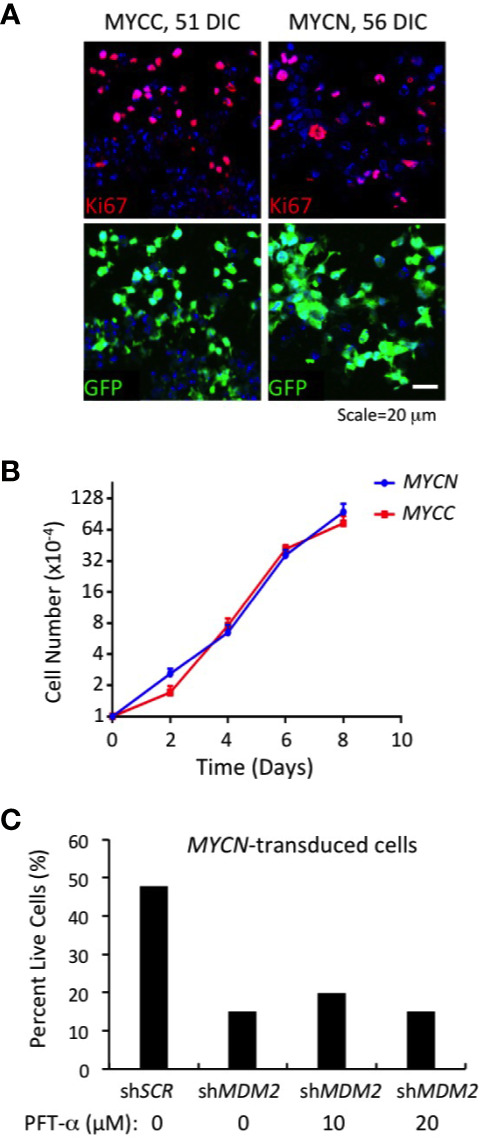
Continuous MDM2-dependent proliferation of *MYC*- and *MYCN-*transformed cone precursors. **(A)** *MYC* and *MYCN*-transduced retinal explants at 51 and 56 DIC and co-stained for Ki67 (top) and GFP+ (bottom). Nuclei are stained with DAPI. **(B)** Growth curves of *MYCN*- and *MYC*-transformed cone precursors after removal from cultured retina, expansion for 30 DIC, and plating at 1×10^4^ cells per well, and measured in three independent wells for each time point. **(C)** Percent live cells detected 78 h after transduction of *MYCN*-transformed cone precursors with sh*MDM2* and control sh*SCR* shRNAs in absence or presence of 10 or 20 µM pifithrin-α (PFT-α) for the final 40 h of culture.

*MYCN*- and *MYC*-transduced cone precursors exhibited continuous proliferation, with most if not all GFP+ cells in high MYCN- or MYC-expressing regions staining positively for Ki67 at 56 DIC or 51 DIC, respectively (**Figure 5A**). *MYCN*-transduced cone precursors also appeared to be oncogenically transformed as they produced tumors resembling human *MYCN-*amplified retinoblastomas in subretinal xenografts (Singh et al., *in preparation*). To evaluate the growth properties and MDM2-dependence of *MYCN*- and *MYC*-transduced cone precursors, GFP+ foci were separated from cultured retinae at 18 DIC by gentle pipetting and established in suspension culture. The cultures grew continuously for more than two months. After the first 30 days in culture, we compared the *MYCN*- and *MYC*-transduced cell growth and observed that the two lines grew at similar rates (**Figure 5B**). Immunostaining and quantitative imaging of PFA-fixed cultured cells in parallel with similarly fixed retinal sections revealed that the cultured cells expressed MDM2 at levels similar to that of *MYCN*- and *MYC*-transduced cone precursors in intact retinae (**Figure 4E**).

To assess whether MDM2 upregulation was needed to sustain proliferation of the *MYCN* and *MYC*-transformed cone precursors, we performed MDM2 knockdown using the same MDM2-directed shRNA vectors as used in established cell lines (**Figures 1****–****3**). After puromycin selection and a total of 78 h after transduction, we noted extensive cell death in sh*MDM2*-transduced *MYCN*- and *MYC*-transformed cells as compared to the control sh*SCR* transduced cells, which was quantified in the case of the *MYCN-*transduced cultures (**Figure 5C**). Cell death was not mitigated by treatment with the p53-inhibitor, pifithrin-α for the final 42 h, suggesting that the cell death was at least in part p53-independent. We conclude that the high MDM2 levels that were induced by MYCN overexpression were critical to the MYCN-transformed cone precursor viability.

## Discussion

This study provides insights into an oncogenic positive feedback circuit in MYCN-driven neural and neuroendocrine cancers. Extending prior observations in *MYCN*-amplified or MYCN-overexpressing neuroblastoma and retinoblastoma cells (15, 17), we show that intrinsically high MDM2 sustains MYCN expression in *MYCN*-amplified SCLC cells but has little or no effect on MYC levels in retinoblastoma, neuroblastoma, SCLC, or medulloblastoma cells. The regulatory specificity was best illustrated in retinoblastoma cell line RB176, in which MYCN and MYC are co-expressed yet MDM2 was needed to sustain only MYCN. MDM2 was also needed to sustain MYCN in two *TP53*-mutant SCLC cell lines, in keeping with the critical role of MDM2 in *TP53*-mutant as well as wild type neuroblastoma and retinoblastoma cells. Among 10 high-MYCN-expressing cell lines examined in this and other studies (15, 17), including five retinoblastoma, three neuroblastoma, and two SCLC lines, all required high-level MDM2 expression for MYCN expression and viability. In two neuroblastoma and two retinoblastoma lines, the mechanism involved upregulation of *MYCN* RNA expression and translation, mediated by MDM2 interaction with the RNA in the neuroblastoma context (15, 17). These findings raise the possibility that most if not all high-MYCN-driven cancers depend on this MDM2 function although it is yet to be proven in some cancer cell types.

At present the basis for MDM2’s selective regulation of MYCN is unclear. The requirement for MDM2 to sustain high MYCN but not MYC in RB176 implies that *MYCN cis*-acting elements have a critical role. A prior study attributed the MDM2 effect to its binding and inhibiting an *MYCN* 3’ UTR AU-rich element that mediates RNA instability (15). However, the *MYC* 3’ UTR has similar AU-rich instability elements (42), suggesting that MDM2 interactions with *MYCN*-specific AU sequences or RNA binding factors may be important. Of note, we found that in SK-N-BE(1) cells, expression of a Flag-tagged MYCN protein encoded by a *MYCN* mini gene retaining nearly all UTR sequences did not decline in response to MDM2 knockdown, whereas the endogenous MYCN was downregulated, suggesting that contextual features beyond *MYCN* UTR sequences contribute to MYCN regulation. Further understanding of the underlying mechanism could reveal a therapeutic target for MYCN-driven cancers.

This study also provides insights into the reciprocal relationship in which MYCN upregulates MDM2. Past studies showed that MYCN upregulates *MDM2* RNA in neuroblastoma cells by binding to *MDM2* promoter E-box sequences (20), and we here corroborate that ectopic MYCN upregulates MDM2 in MYC-expressing SH-SY5Y. Conversely, MYCN KD was found to downregulate MDM2 in IMR-32 neuroblastoma cells (20) but not in NB-1691 and NB-1643 neuroblastoma or RB176 retinoblastoma cells (16, 17), implying that MYCN dependent MDM2 expression is cell line-specific. However, the relevance of the MYCN → MDM2 axis in developing tumors, as opposed to immortalized cancer cell lines, had not been demonstrated. Here, we show that MYCN overexpression upregulates MDM2 in a fetal retina model that recapitulates features of human *MYCN*-amplified retinoblastoma. The rapid induction of MDM2 suggests that high MYCN protein levels suffice to induce MDM2 and initiate tumorigenesis in the absence of other genetic or epigenetic alterations. MYCN’s ability to induce MDM2 in cone precursors contrasts with its failure to upregulate MDM2 in sympathetic ganglion cells that initiate tumorigenesis in the *TH-MYCN* transgenic mouse neuroblastoma model, where MYCN engaged an alternative BMI1-mediated resistance to p53 signaling (43). To our knowledge the MYCN → MDM2 axis has not been observed in animal models and thus may be human-specific, with important implications for modeling MYCN-driven cancers.

Additionally, we found that MYC, as well as MYCN, upregulated MDM2 and induced cone precursor cell cycle entry and robust proliferation. Although MYC was suggested to drive MDM2 expression based on its association with the *MDM2* promoter in B cell lymphoma cells (44), to our knowledge, this is the first demonstration that MYC overexpression upregulates MDM2 protein. The MYC- and MYCN-transformed cone precursors’ sensitivity to MDM2 knockdown suggests that MDM2 upregulation is an important part of MYC- as well as MYCN-mediated cancer initiation.

The ectopic MYCN-mediated upregulation of MDM2 raises the possibility that this circuitry is also engaged during normal development. In the retina, MDM2 is normally expressed at higher levels in maturing cone precursors than in other retinal cell types (18, 21). One factor that may contribute to MDM2 upregulation is the cone lineage transcription factor RXRγ, which binds to RXRγ response elements in the human *MDM2* promoter (18). However, RXRγ likely does not suffice to upregulate MDM2, as it is expressed at similar levels in immature and maturing cones (45). Additionally, MYCN may contribute to developmental MDM2 expression, as MYCN is expressed at higher levels in cone precursors than in other retinal cell types and increases prior to MDM2 during cone maturation [**Figure 4Di** and ref (18)]. A key factor in this process may be the MYCN-induced WDR5, which collaborates with MYCN at the MDM2 promoter (46). Another candidate co-regulator is TEAD4, which drives the MYCN-induced transcription network in *MYCN*-amplified neuroblastomas (47), although we were unable to detect TEAD4 in a MYCN-transduced fetal retina.

Our finding that MYCN promotes MDM2 expression in the retinoblastoma cell of origin and that MDM2 promotes MYCN expression in retinoblastoma cells suggests that MYCN and MDM2 comprise a positive feedback circuit that initiates at the onset of MYCN-driven retinoblastoma genesis. Nevertheless, while we detected MYCN-induced upregulation of MDM2, we did not observe the reciprocal MDM2-induced upregulation of the ectopic MYCN in cone transduced precursors, in keeping with the *MYCN* expression vector’s lack of *MYCN* UTR sequences that are implicated in the MDM2 response (15). Instead, the high-level MYCN protein corresponded to its high RNA expression from the *BE-GFP* vector (**Figure 4B**). The vector’s high expression in cone precursors relative to other retinal cells suggests that these cells are unusually prone to robustly activate the vector’s *EF1a* enhancer, perhaps in conjunction with the ectopic MYCN. Sustained ectopic MYCN expression may suffice to induce oncogenic transformation in this setting, whereas MDM2-mediated positive feedback, to further upregulate MYCN translation, is needed for development of intrinsically MYCN-driven neural and neuroendocrine tumors.

Although MYCN-induced upregulation of MDM2 was not needed to reciprocally sustain ectopic MYCN levels in our system, it was nevertheless required for MYCN-transformed cone precursor proliferation and survival (**Figure 5**). One likely role of the increased MDM2 expression was to suppress p53 activity. However, this is unlikely to be the only important function, as cell death was not abrogated by p53 inhibitor pifithrin-α and as MDM2 is critical to retinoblastoma cell survival via p53-independent mechanisms beyond MYCN induction (17). MDM2 has extensive p53-independent functions whose context-specific roles are yet to be defined (48). Identifying these roles in human cancer-originating cells may provide opportunities to interfere with the genesis and propagation of MYCN-driven neural and neuroendocrine cancers.

## Data Availability Statement

The original contributions presented in the study are included in the article/**Supplementary Material**. Further inquiries can be directed to the corresponding author.

## Ethics Statement

The studies involving human participants were reviewed and approved by Institutional Review Boards of University of Southern California and Children’s Hospital Los Angeles. The patients/participants provided their written informed consent to participate in this study.

## Author Contributions

Conception and design of the study: HT, HS, D-LQ, and DC. Performed experiments: HT, HS, WG, LC, LR, D-LQ. Discussions and provision of medulloblastoma cell lines: GS and AE-E. Supervision: HS, LC, D-LQ, DC. Writing and preparation of manuscript: HT HS, LC, and DC. All authors contributed to the article and approved the submitted version.

## Funding

This study was supported by an NIH Ruth L. Kirschstein National Research Service Award Institutional Research Training Grant 2T32CA009659-24 to Y. DeClerck (HT), a Saban Research Institute Research Career Development Fellowship (DQ), the Nautica Malibu Triathlon event produced by MESP Inc. (HT, DC), the Rachel Ann Hage Neuro-oncology Fund and Harriet H. Samuelsson Foundation (AE-E), and NIH grant R01CA137124 (DC).

## Conflict of Interest

The authors declare that the research was conducted in the absence of any commercial or financial relationships that could be construed as a potential conflict of interest.
