# Supplementary material for: Reciprocal Induction of MDM2 and MYCN in Neural and Neuroendocrine Cancers
Source: Front Oncol. 2020 Dec 23;10:563156. doi: 10.3389/fonc.2020.563156 (PMC7793692; doi:10.3389/fonc.2020.563156)
Supplement: Supplementary file 1 [file Table_1.docx]

**Tran H., Singh HP. *et al*., Reciprocal Induction of MDM2 and MYCN in Neural and Neuroendocrine Cancers.**

**Supplementary Table 1.**

**Table S1. *In situ* HCR probe sequences**

| MYCN probes with linkers and initiators (B2 system-Alexa-594) | |
| --- | --- |
| **Probe Name** | **Sequence** |
| MYCN_1 | tctgggttcttgcagatcatAAAAAGCTCAGTCCATCCTCGTAAATCCTCATCAATCATC |
| MYCN_2 | agggctgtagcgagtcaaacAAAAAGCTCAGTCCATCCTCGTAAATCCTCATCAATCATC |
| MYCN_3 | gccgaagtagaagtcatcttAAAAAGCTCAGTCCATCCTCGTAAATCCTCATCAATCATC |
| MYCN_4 | gctcaaacttcttccagatgAAAAAGCTCAGTCCATCCTCGTAAATCCTCATCAATCATC |
| MYCN_5 | tcgttctcaagcagcatctcAAAAAGCTCAGTCCATCCTCGTAAATCCTCATCAATCATC |
| MYCN_6 | gttcacgggaaaggggaagaAAAAAGCTCAGTCCATCCTCGTAAATCCTCATCAATCATC |
| MYCN_7 | catcgtttgaggatcagctcAAAAAGCTCAGTCCATCCTCGTAAATCCTCATCAATCATC |
| MYCN_8 | tctttatcttcttctgtgggAAAAAGCTCAGTCCATCCTCGTAAATCCTCATCAATCATC |
| MYCN_9 | gatgacactcttgagcggacAAAAAGCTCAGTCCATCCTCGTAAATCCTCATCAATCATC |
| MYCN_10 | cagagtttcgggggctcaagAAAAAGCTCAGTCCATCCTCGTAAATCCTCATCAATCATC |
| MYCN_11 | gatgttgtggtttctgcgacAAAAAGCTCAGTCCATCCTCGTAAATCCTCATCAATCATC |
| MYCN_12 | tgagcgtgagaaagctggacAAAAAGCTCAGTCCATCCTCGTAAATCCTCATCAATCATC |
| MYCN_13 | ccttctcattctttaccaacAAAAAGCTCAGTCCATCCTCGTAAATCCTCATCAATCATC |
| MYCN_14 | gagggagtggacatactcagAAAAAGCTCAGTCCATCCTCGTAAATCCTCATCAATCATC |
| MYCN_15 | ttttctttagcaactgctgcAAAAAGCTCAGTCCATCCTCGTAAATCCTCATCAATCATC |
| MYCN_16 | tgagaagcgtctagcaagtcAAAAAGCTCAGTCCATCCTCGTAAATCCTCATCAATCATC |

| WPRE probes with linkers and initiators (B1 system-Alexa-647) | |
| --- | --- |
| **Probe Name** | **Sequence** |
| WPRE_1 | gtaatccagaggttgattgtTATAGCATTCTTTCTTGAGGAGGGCAGCAAACGGGAAGAG |
| WPRE_2 | ataccagtcaatctttcacaTATAGCATTCTTTCTTGAGGAGGGCAGCAAACGGGAAGAG |
| WPRE_3 | gcgtatccacatagcgtaaaTATAGCATTCTTTCTTGAGGAGGGCAGCAAACGGGAAGAG |
| WPRE_4 | agccatacgggaagcaatagTATAGCATTCTTTCTTGAGGAGGGCAGCAAACGGGAAGAG |
| WPRE_5 | gccacaactcctcataaagaTATAGCATTCTTTCTTGAGGAGGGCAGCAAACGGGAAGAG |
| WPRE_6 | ttgcgtcagcaaacacagtgTATAGCATTCTTTCTTGAGGAGGGCAGCAAACGGGAAGAG |
| WPRE_7 | aaaggagctgacaggtggtgTATAGCATTCTTTCTTGAGGAGGGCAGCAAACGGGAAGAG |
| WPRE_8 | atagggagggggaaagcgaaTATAGCATTCTTTCTTGAGGAGGGCAGCAAACGGGAAGAG |
| WPRE_9 | aacaccacggaattgtcagtTATAGCATTCTTTCTTGAGGAGGGCAGCAAACGGGAAGAG |
| WPRE_10 | catggaaaggacgtcagcttTATAGCATTCTTTCTTGAGGAGGGCAGCAAACGGGAAGAG |
| WPRE_11 | gaatccaggtggcaacacagTATAGCATTCTTTCTTGAGGAGGGCAGCAAACGGGAAGAG |
| WPRE_12 | gaagggacgtagcagaaggaTATAGCATTCTTTCTTGAGGAGGGCAGCAAACGGGAAGAG |
| WPRE_13 | aaggcgaagacgcggaagagTATAGCATTCTTTCTTGAGGAGGGCAGCAAACGGGAAGAG |
| WPRE_14 | aaagggagatccgactcgtcTATAGCATTCTTTCTTGAGGAGGGCAGCAAACGGGAAGAG |
| WPRE_15 | aaggtaccgagctcgaattcTATAGCATTCTTTCTTGAGGAGGGCAGCAAACGGGAAGAG |
